# Construction of a risk stratification model integrating ctDNA to predict response and survival in neoadjuvant-treated breast cancer

**DOI:** 10.1186/s12916-023-03163-4

**Published:** 2023-12-12

**Authors:** Zhaoyun Liu, Bo Yu, Mu Su, Chenxi Yuan, Cuicui Liu, Xinzhao Wang, Xiang Song, Chao Li, Fukai Wang, Jianli Ma, Meng Wu, Dawei Chen, Jinming Yu, Zhiyong Yu

**Affiliations:** 1 Department of Radiation Oncology and Shandong Provincial Key Laboratory of Radiation Oncology, Shandong Cancer Hospital and Institute, Shandong First Medical University and Shandong Academy of Medical Sciences, Jinan, 250117 Shandong China; 2 Breast Cancer Center, Shandong Cancer Hospital and Institute, Shandong First Medical University and Shandong Academy of Medical Sciences, Jinan, 250117 Shandong China; 3 Shandong University Cancer Center, Jinan, 250117 Shandong China; 4 Berry Oncology Institutes, Beijing, China; 5 Yantai Yuhuangding Hospital Affiliated to Qingdao University, Yantai, China; 6 Thyroid & Breast Surgery Department, LiaoCheng Peoples’s Hospital, Liaocheng, 252000 China; 7 Department of Radiation Oncology, Harbin Medical University Cancer Hospital, Harbin, 150081 China; 8 Research Unit of Radiation Oncology, Chinese Academy of Medical Sciences, Jinan, 250117 China

**Keywords:** Breast cancer, Neoadjuvant chemotherapy, pCR, Prediction model, ctDNA

## Abstract

**Background:**

The pathological complete response (pCR) to neoadjuvant chemotherapy (NAC) of breast cancer is closely related to a better prognosis. However, there are no reliable indicators to accurately identify which patients will achieve pCR before surgery, and a model for predicting pCR to NAC is required.

**Methods:**

A total of 269 breast cancer patients in Shandong Cancer Hospital and Liaocheng People’s Hospital receiving anthracycline and taxane-based NAC were prospectively enrolled. Expression profiling using a 457 cancer-related gene sequencing panel (DNA sequencing) covering genes recurrently mutated in breast cancer was carried out on 243 formalin-fixed paraffin-embedded tumor biopsies samples before NAC from 243 patients. The unique personalized panel of nine individual somatic mutation genes from the constructed model was used to detect and analyze ctDNA on 216 blood samples. Blood samples were collected at indicated time points including before chemotherapy initiation, after the 1^st^ NAC and before the 2^nd^ NAC cycle, during intermediate evaluation, and prior to surgery. In this study, we characterized the value of gene profile mutation and circulating tumor DNA (ctDNA) in combination with clinical characteristics in the prediction of pCR before surgery and investigated the prognostic prediction. The median follow-up time for survival analysis was 898 days.

**Results:**

Firstly, we constructed a predictive NAC response model including five single nucleotide variant (SNV) mutations (TP53, SETBP1, PIK3CA, NOTCH4 and MSH2) and four copy number variation (CNV) mutations (FOXP1-gain, EGFR-gain, IL7R-gain, and NFKB1A-gain) in the breast tumor, combined with three clinical factors (luminal A, Her2 and Ki67 status). The tumor prediction model showed good discrimination of chemotherapy sensitivity for pCR and non-pCR with an AUC of 0.871 (95% CI, 0.797–0.927) in the training set, 0.771 (95% CI, 0.649–0.883) in the test set, and 0.726 (95% CI, 0.556–0.865) in an extra test set. This tumor prediction model can also effectively predict the prognosis of disease-free survival (DFS) with an AUC of 0.749 at 1 year and 0.830 at 3 years. We further screened the genes from the tumor prediction model to establish a unique personalized panel consisting of 9 individual somatic mutation genes to detect and analyze ctDNA. It was found that ctDNA positivity decreased with the passage of time during NAC, and ctDNA status can predict NAC response and metastasis recurrence. Finally, we constructed the chemotherapy prediction model combined with the tumor prediction model and pretreatment ctDNA levels, which has a better prediction effect of pCR with the AUC value of 0.961.

**Conclusions:**

In this study, we established a chemotherapy predictive model with a non-invasive tool that is built based on genomic features, ctDNA status, as well as clinical characteristics for predicting pCR to recognize the responders and non-responders to NAC, and also predicting prognosis for DFS in breast cancer. Adding pretreatment ctDNA levels to a model containing gene profile mutation and clinical characteristics significantly improves stratification over the clinical variables alone.

**Supplementary Information:**

The online version contains supplementary material available at 10.1186/s12916-023-03163-4.

## Background

Breast cancer is the most commonly diagnosed cancer in females [1–3]. Neoadjuvant chemotherapy (NAC) has long been considered the preferred treatment approach for locally advanced breast cancer to downstage the tumor while concurrently allowing for in vivo assessment of tumor response to NAC [4]. Despite impressive successes, approximately 10% of breast cancer patients with no response fails to benefit from NAC [5]. These patients could benefit from stopping NAC and proceeding directly to surgery or switching to a different treatment. Therefore, assessing the sensitivity of patients to NAC is an important task in clinical practice.

Pathological complete response (pCR) by examination of surgical specimens after NAC is associated with long-term survival and has been used as the primary endpoint of neoadjuvant trials [6, 7]. A treatment monitoring biomarker that can accurately predict pCR before the surgery is required. Clinically, imaging evaluations, particularly ultrasound, mammography, and breast magnetic resonance imaging (MRI) are mainly used to evaluate the extension of the mass, which the disease progression has occurred [8, 9]. Therefore, more sensitive biomarkers are needed that will earlier detection of progression to identify the response to NAC.

Some polygenic predictor panels have been developed to predict the pCR to NAC and guide physicians to make adjuvant treatment decisions [10, 11]. Huang Liang et al. developed a predictor of the pCR in triple-negative breast cancer (TNBC) patients with DNA repair genes to NAC [12]. Masanori Osh et al. developed a five-gene score model to predict the pCR to NAC for estrogen receptor-positive/Her2-negative breast cancer and a novel three-gene score as a predictive biomarker for pCR after NAC in TNBC [10, 13]. However, these single-platform profiling markers fail to accurately predict the response to NAC with the complexity of the tumor ecosystem and the dynamic variability of treatment. Undoubtedly, clinicians continue to select NAC patients by clinical experience.

Circulating tumor DNA (ctDNA) corresponds to the DNA fragments released into the blood by the tumor, and utilizing ctDNA to track tumor progression has great potential for the clinical treatment [14]. ctDNA levels have been shown to be correlated with tumor loading to predict therapeutic prognosis and survival in pan-cancer [15, 16]. Chaudhuri et al. reported that ctDNA was detected in patients with disease recurrence 5.2 months earlier than imaging evaluations, offering therapeutic opportunities to treat patients while tumor burden and heterogeneity are at their lowest in lung cancer [17]. Continuous measurement of ctDNA is a potential method for early tumor surveillance, and it serves as an objective parameter for treatment response and earlier relapse detection.

Therefore, we conducted a study to establish a risk model based on the gene mutation profile and clinical characteristics integrating ctDNA dynamic monitoring of breast cancer patients treated with NAC to develop a predictable NAC response for breast cancer patients.

## Methods

### Patient eligibility

Approval for this prospective study was obtained from the Human Research Ethics Committee of Shandong Cancer Hospital (SDTHEC201802002) and was registered at clinicaltrial.gov (clinical trial No. NCT03688035). All patients provided written informed consent. From January 2016 to April 2020, a total of 269 female breast cancer patients who received NAC were enrolled, and 246 enrolled patients were eligible. Patients were excluded (*n* = 23) if they were defined with distant metastasis before NAC, failed to complete the chemotherapy regimen, or missed the clinical date. All patients were staged according to the American Joint Committee on Cancer (AJCC) 8th edition TNM staging guideline system. All of the patients received 4–8 cycles of NAC with anthracycline and taxane-based regimens, and all Her2-positive patients received anti-Her2 regimens including trastuzumab or dual Her2 blockade with trastuzumab plus pertuzumab before surgical resection of the tumor.

Miller-Payne (MP) grading and residual cancer burden (RCB) index were used to validate the response to NAC. The RCB evaluation system (www.mdanderson.org/breastCancer_RCB) was used to calculate the RCB index [18]. pCR (stage yp-T0/is, ypN0) was defined as RCB = 0, and residual disease (no-pCR) was placed into three predefined subgroups (RCB-I, RCB-II, and RCB-III) on the basis of predefined cut-off points of 1.36 and 3.28 index scores. RCB and pCR status were evaluated by two independent pathologists. If their conclusions were inconsistent, a third pathologist reassessed the situation.

### Sample collection

**Fig. 1:**
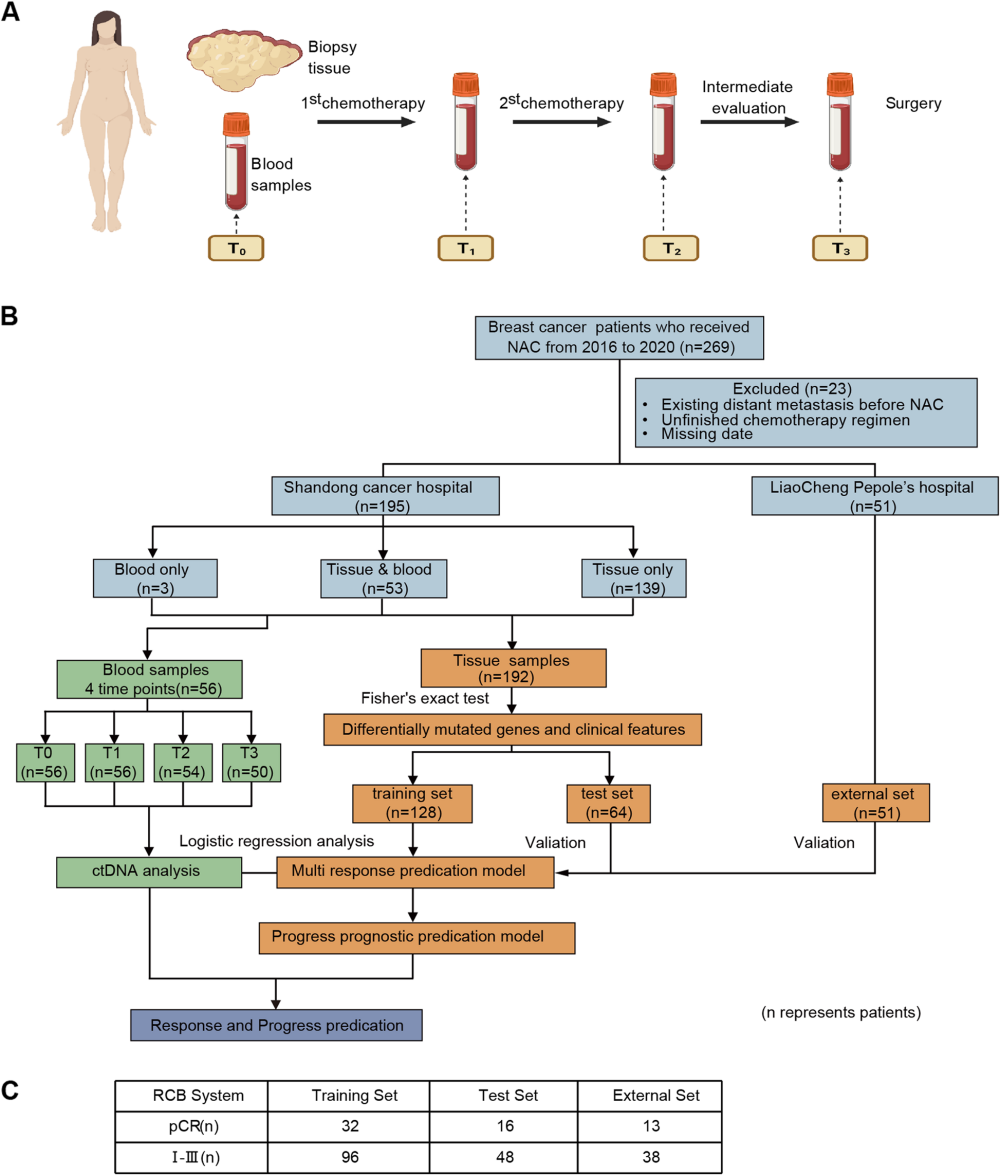
The study design, sample collections, and patients’ response. **A** Sample collection at different points. **B** Condition of enrolled patients. **C** Number of patients in different groups responding to pCR

A total of 243 tissue puncture samples from formalin fixation and paraffin embedding (FFPE) were obtained before the initiation of chemotherapy by core needle biopsy from 246 patients. Among the 246 patients, 56 patients underwent ctDNA testing of blood samples. The blood samples were collected from each patient dynamically over the course of NAC at four time points as follows: before NAC (T_0_), after the 1^st^ NAC and before the 2^nd^ NAC cycle (T_1_), during intermediate evaluation (T_2_), and after the end of NAC but before surgery (T_3_) (Fig. 1A). The baseline plasma samples of all 56 patients were collected, while six patients failed to complete the sample collection at all four time points, and the remaining 50 patients completed the entire plasma sample collection process (Fig. 1B).

### Tissue and plasma DNA preparation and genomic DNA extraction

A GeneRead DNA FFPE kit (Qiagen, USA) was used to extract genomic DNA (gDNA) from FFPE and fresh tissue samples, and a DNA blood Midi/Mini kit (Qiagen, USA) was used to extract genomic DNA from white blood cell samples according to the manufacturer's instructions. The MagMAX cell-free DNA Isolation Kit (Thermo Company) was used to separate plasma cell-free DNA (cfDNA). The quality of purified DNA was quantified by gel electrophoresis and using a Qubit® 4.0 fluorometer (Life Technologies, USA).

### Construction of the NGS gene panel sequencing library based on gDNA and cfDNA

First, the purified gDNA was fragmented to approximately 200 bp by enzymatic hydrolysis (5X WGS Fragmentation Mix, Qiagen, USA). After end repair and A-tail connection, the two ends were connected with T-adaptors and then PCR amplified to form a prelibrary. The purified prelibrary was hybridized with a customized biotin probe pool (457 gene panel, Berry Oncology, Beijing, China) to capture the target clip. According to the manufacturer’s instructions, the 96 rxn xGen Exome Research Group v1.0 (Integrated DNA Technologies, USA) was used to prepare the final sequencing library.

For targeted sequencing of cfDNA, a prelibrary was prepared according to the method described above. Internally designed panels were used to capture cfDNA fragments and generate sequencing libraries. The sequencing library was applied to the NovaSeq 6000 platform (Illumina, San Diego, USA) in 150PE mode.

The generated sequence was trimmed, low-quality filtered, and subjected to variant calls. Variations were filtered into nonsynonymous SNP, indel, and splicing variations. For gDNA, somatic mutations were left with allele frequencies (VAF) ≥ 3%, and cancer hotspots were retained with VAF ≥ 1%. For cfDNA, the frequency of variant alleles (cut-off value ≥ 0.5%) was used to identify somatic mutations, the frequency of variant alleles (cut-off value ≤ 0.1%) was used to screen for cancer hot spots, and at least 20 high-quality reads were screened.

### Bioinformatics analysis of gene mutations

FASTP [19] was used to trim adapters and delete low-quality sequences to obtain clean reads. The clean reads were compared with the Ensemble GRCh37/hg19 reference genome executed by BWA. The PCR repetitive sequence was processed to consensus sequence by GenCore [20], then SAMtools [21] was used to detect somatic single nucleotide variants (SNVs), insertions and deletions (InDels), and then false variants were filtered by a series of methods such as vaf cutoff, paired control sample, negative background database. HGVS variant description was annotated with ANNOVAR software [22]. After annotation, we used PopFreqMax > 0.05 to eliminate SNVs and InDels, then retained nonsynonymous SNVs and InDels with VAF > 0.5% or VAF > 0.1% among the cancer hot spots in the patient database for further analysis. Somatic copy number variants (CNVs) were called by CNVkit [23] through several steps, such as depth normalization and GC correction. If the copy number > 3, we marked the target as a target gain, and if the copy number < 1, we marked the target as a target loss. The tumor mutation burden (TMB) was defined as the number of all nonsynonymous mutations and indel variants per megabase of coding regions.

### Statistical analysis

Baseline characteristics analysis was performed on all 246 patients, including the distribution of patients with baseline genomic or clinical characteristics in the pCR/non-pCR group and the correlation between baseline characteristics and pCR status or patient prognosis. The association analysis between mutation detection and prognosis was performed on 56 patients with continuous ctDNA test data (that is, the completion of the entire study).

Fisher’s exact test (two-sided test) was used to analyze the impact of baseline pathological characteristics (such as breast cancer type, Ki67, age, sex, and disease stage) and mutation characteristics on non-pCR. Those features related to the pCR/non-pCR state were selected to construct a predictive model for pCR/non-pCR prediction. Usually, we chose to perform the next step of data analysis with Fisher's exact test (*P* value < 0.05) for the feature. A total of 192 patients who had tissue samples from Shandong Cancer Hospital were randomized into a training cohort (*n* = 128) and a testing cohort (*n* = 64) using the “caret” R package. The training cohort was used to find a meaningful signature, and the testing cohort and patients of Liaocheng People’s Hospital (*n* = 51) were used to validate its efficiency.

In the training cohort, significant SNVs and CNVs with a mutation frequency greater than 10% including five SNVs and 10 CNVs mutations were subjected to multivariate stepwise logistic regression analysis. The nine mutated genes including five SNV mutations and four CNV mutations were identified by step-by-step logistic regression analysis. Then, a multi-response model was built including only clinical characteristics, only SNV characteristics, only CNV characteristics, both SNV and CNV characteristics, both SNV and clinical characteristics, both CNV and clinical characteristics, and SNV, CNV, and clinical characteristics for a total of 6 response models. All models were based on the random forest method and developed in a tenfold cross-validation (CV) schema. Performance was assessed in terms of accuracy (ACC), sensitivity, and specificity by receiver operating characteristic (ROC) curve analysis. A nomogram was also applied to estimate the performance of the signature. ROC analysis was performed using the “caret” and “ROCR” R packages and a nomogram was generated using the “rms” R package.

We defined disease-free survival (DFS) as the time from the breast surgery until disease progression (including local or distant recurrences) or death. We constructed a DFS prediction model based on the signature of the best model among the multiresponse models for 192 patients from Shangdong Cancer Hospital in the training set and test set. Then, a risk score was calculated for each patient, and patients were divided into high- and low-risk groups based on the median risk score. The ROC analysis was performed using the “timeROC” R package. Kaplan–Meier curves were generated for survival analysis, and the log-rank test was used for comparisons.

The threshold for ctDNA positivity was determined by the mutation numbers of nine genes included in the conducted tumor prediction model. If there were more than two mutations among these nine genes, then this sample was recognized as ctDNA positive [24]. ctDNA fractions were calculated as 2/(1/Max(VAF) + 1) [25]. One-way analysis of variance (ANOVA) was applied for comparison of ctDNA fractions in different groups. Use the random forest model to obtain the probability of treatment response or non-response for each sample, and combine the ctDNA status at different time points with the random forest model to construct a combined tissue and blood efficacy prediction model.

Conduct multivariable Cox regression analysis using the mutation levels of the nine genes at the tissue level, clinical features, and the ctDNA status at each time point to obtain the risk scores for each sample, and construct a DFS prediction model. Similarly, divide the samples into high and low-risk groups based on the median risk score.

A flowchart for the algorithm is shown in sFig. 4. All statistical analyses were performed using R software, version 3.6.3 (www.r-project.org). For all statistical analyses, *p* < 0.05 was considered statistically significant.

## Results

### Patient characteristics

A total of 192 patients who had tissue samples in Shandong Cancer Hospital and 51 patients in Liaocheng People’s Hospital were used to predict the residual cancer burden status of patients receiving NAC. The specific clinical information is shown in Table 1. The median age was 50 years old, and 59.3% were younger than 50 years old. Most patients were in stage III (51.9%). The subtypes of Her2 positivity, luminal A, luminal B (Her2-), luminal B (Her2 +), and TNBC accounted for 19.3% (47/243), 21.4% (52/243), 23.5% (57/243), 18.1% (44/243), and 17.7% (43/243), respectively. The pCR rate in different molecular subtypes was shown in Additional file 1: Table S1. Patients with Ki67 expression > 20% accounted for the main population (67.8%). Postoperative pathological examination showed that 61 patients (25.1%) had achieved pCR by RCB index. For those patients who showed non-pCR, 11.9% (29/243), 35.8% (87/243) and 27.2% (66/243) reached RCB-I, RCB-II and RCB-III, respectively. 87.2% of patients received the anthracyclines plus taxanes-based chemotherapy, 5.8% received the anthracycline-containing regimen only, and 7% of patients received taxanes-containing regimen alone (Additional file 1: Table S2 and Table S3). An overview of the study design was shown in Fig. 1.

**Table 1:** Patient clinical characteristics

	**Level**	**Overall**	**Training cases**	**Validation cases**	**External validation cases**
Number		243	128	64	51
Patient age (%)	Younger (≤ 50)	144 (59.3)	79 (61.7)	36 (56.2)	29 (56.9)
	Older (> 50)	99 (40.7)	49 (38.3)	28 (43.8)	22 (43.1)
Tumor stage (%)	Stage I–II	117 (48.1)	51 (39.8)	25 (39.1)	41 (80.4)
	Stage III	126 (51.9)	77 (60.2)	39 (60.9)	10 (19.6)
Mulecular subtypes (%)	Her2 +	47 (19.3)	28 (21.9)	10 (15.6)	9 (17.6)
	luminal A	52 (21.4)	21 (16.4)	12 (18.8)	19 (37.3)
	luminal B (Her2 −)	57 (23.5)	32 (25.0)	20 (31.2)	5 (9.8)
	luminal B (Her2 +)	44 (18.1)	28 (21.9)	13 (20.3)	3 (5.9)
	TNBC	43 (17.7)	19 (14.8)	9 (14.1)	15 (29.4)
Ki67 expression (%)^a^	≤ 20%	78 (32.2)	34 (26.8)	20 (31.2)	24 (47.1)
	> 20%	164 (67.8)	93 (73.2)	44 (68.8)	27 (52.9)
Menstrual status (%)	Postmenopausal	149 (61.3)	85 (66.4)	40 (62.5)	24 (47.1)
	Premenopausal	94 (38.7)	43 (33.6)	24 (37.5)	27 (52.9)
Response to NAC, MP (%)	1	18 (7.4)	8 (6.2)	5 (7.8)	5 (9.8)
	2	52 (21.4)	25 (19.5)	13 (20.3)	14 (27.5)
	3	51 (21.0)	25 (19.5)	15 (23.4)	11 (21.6)
	4	41 (16.9)	27 (21.1)	11 (17.2)	3 (5.9)
	5	81 (33.3)	43 (33.6)	20 (31.2)	18 (35.3)
Response to NAC, RCB (%)	pCR	61 (25.1)	32 (25.0)	16 (25.0)	13 (25.5)
	RCB I	29 (11.9)	17 (13.3)	8 (12.5)	4 (7.8)
	RCB II	87 (35.8)	49 (38.3)	21 (32.8)	17 (33.3)
	RCB III	66 (27.2)	30 (23.4)	19 (29.7)	17 (33.3)
Treatment regimen (%)	EC	14 (5.8)	6 (4.7)	7 (10.9)	1 (2.0)
	EC-T (H/HP), T(H)-EC, TEC	212 (87.2)	112 (87.5)	56 (87.5)	44 (86.3)
	T(HP), TC(H), TCbH(P)	17 (7.0)	10 (7.8)	1 (1.6)	6 (11.8)

*Abbreviation*: *E*, anthracycline; *C*, cyclophosphamide; *T,* taxane; *H*, trastuzumab; *P*, pertuzumab; *Cb*, platinum

^a^Ki67 data was missing for 1 patient

### Somatic mutation detection in tissue samples

**Fig. 2:**
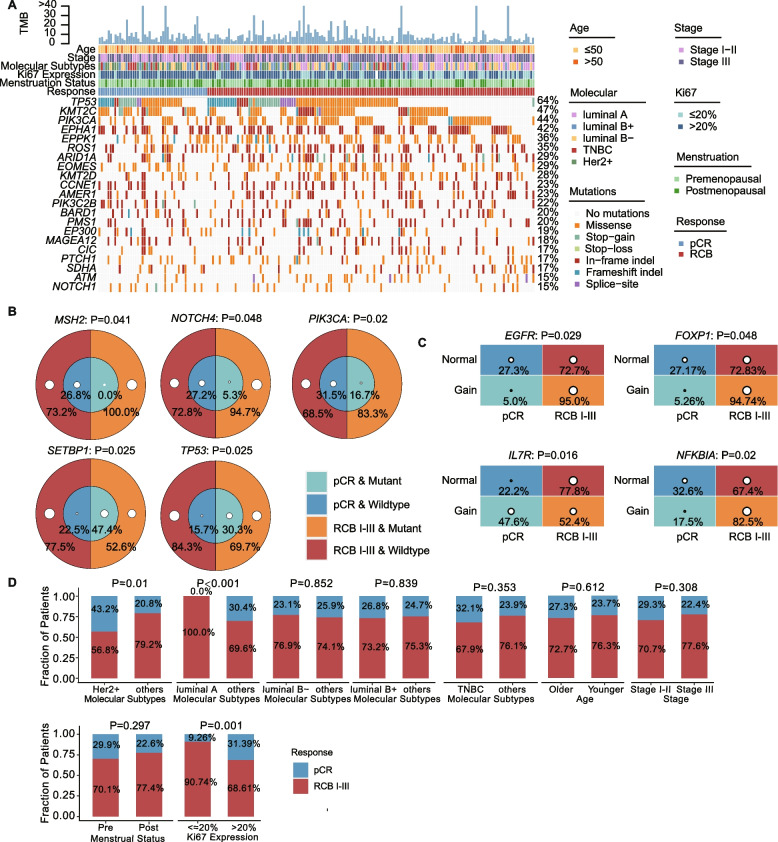
The landscape of clinical and mutational characteristics. **A** The landscape of highly mutated genes. **B** Significantly different SNV included in the model in different chemotherapy responses. **C** Significantly different CNV included in the model in different chemotherapy responses. **D** Comparing the differences in clinical characteristics of different chemotherapy responses. *P* values were calculated using Fisher’s exact test. The size of the white dots in **B** and **C** represents the size of the samples

To discover somatic variations in tissue samples used for model construction, we extracted DNA from FFPE samples of punctured tissue samples and performed NGS-based 457 gene panel testing. We analyzed and summarized the somatic mutations of 192 samples with high-frequency mutation ≥ 10%. The 425 unique genes were identified, and the top five highly mutated genes were TP53, KMT2C, PIK3CA, EPHA1 and EPPK1 with mutation frequencies of 64% (123/192), 47% (90/192), 44% (84/192), 42% (80/192), and 36% (69/192) (Fig. 2A and Additional file 1: Table S4). The results of somatic CNV showed that 114 genes in tumor tissues were amplified with at least three times of normal tissues. There were 43 genes that were deleted, and 200 genes were both amplified and deleted (Additional file 1: Table S5).

### Identified the features significantly associated with pCR

We investigated the relationship with pCR and gene mutation characteristics and clinical phenotypes. All 192 patients were randomly divided into two groups in a 2:1 ratio into a training set (*n* = 128) and a test set (*n* = 64). In the training set, we first performed differential mutation gene analysis on pCR and no-pCR (RCB-I, RCB-II, and RCB-III) patients. Using Fisher’s exact test, we found five different SNV mutated genes (TP53, SETBP1, PIK3CA, NOTCH4, and MSH2) and ten CNV mutated genes (B4GALT3-gain, CDK12-gain, EGFR-gain, PRDM1-gain, GATA2-loss, GNA11-loss, NOTCH3-loss, FOXP1-gain, IL7R-gain, and NFKB1A-gain) with a mutation frequency greater than 10% (Additional file 1: Table S6). To further screen for the factors used to predict pCR status, stepwise logistic regression was used to screen for the differentially mutated genes, and nine differentially mutated genes that were significantly related to the pCR status (MSH2, NOTCH4, PIK3CA, SETBP1, TP53, EGFR, FOXP1, IL7R, NFKB1A) were screened (Fig. 2B, C). Given the potential importance of clinical factors in NAC, we screened the clinically relevant factors of luminal A, Her2^+^, and Ki67 related to NAC using Fisher’s exact test. Patients with luminal A were less sensitive to NAC than patients with other subtypes (*P* < 0.001) and no luminal A patients achieved pCR. In Her2^+^ patients, the proportion of patients who achieved pCR after NAC was significantly higher than that of non-Her2^+^ patients (43.2% vs 20.8%, *P* = 0.01). In addition, patients with higher Ki67 expression pretherapy had a higher proportion of attained pCR patients (31.39% vs 9.26%, *P* = 0.001) (Fig. 2D).

### Machine learning integrates the gene mutation status and clinical factors to build a tumor prediction model to predict the NAC response

**Fig. 3:**
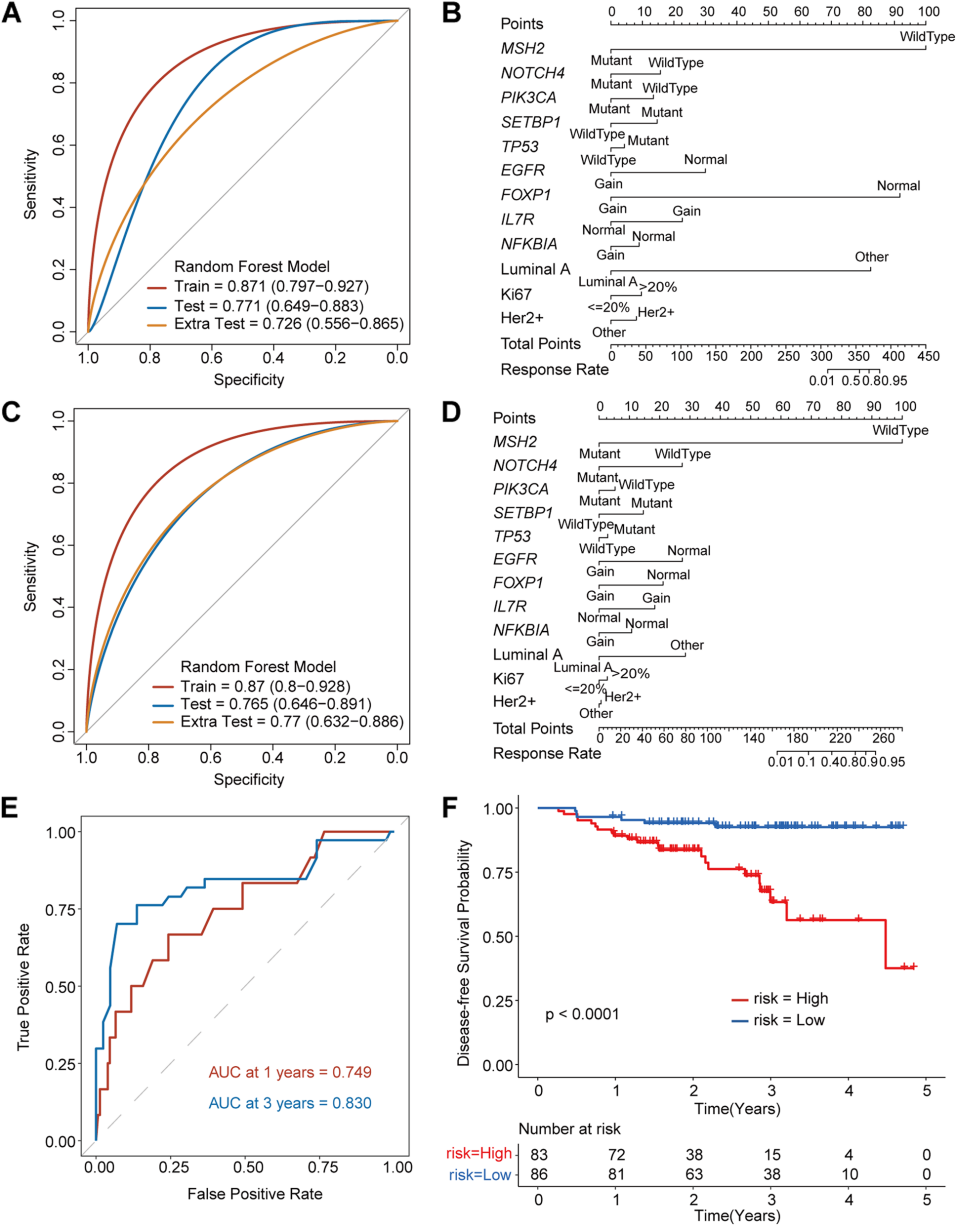
Predicting response and DFS combing mutation characteristics and clinical characteristics. **A** The ROC curve of predictive model in RCB index system. **B** Nomogram from stepwise logistic regression for predicting pCR in RCB index system. **C** The ROC curve of predictive model in MP scoring system. **D** Nomogram from stepwise logistic regression for predicting pCR in MP scoring system. **E** Predicting DFS combing important mutation and clinical characteristics. **F** Kaplan–Meier curves for patients in high- and low-risk groups. Response rate refers to the probability of a patient responding to treatment

Above, the nine mutant genes (five SNV mutations and four CNV mutations) and three clinical factors were identified that were associated with response to NAC. This motivated the use of a machine learning framework to integrate these factors into a predictive model of pCR. We investigated a number of prediction models including the gene mutation information and clinical factors alone and the combination of mutation information and clinical factors. The results found that a combination of nine mutant genes and three clinical factor models had the higher sensitivity and specificity than other combinations (Additional file 2: Fig. S1) in the training test (AUC: 0.871, 95% CI: 0.797–0.927), in the verification set (AUC: 0.771, 95% CI: 0.649–0.883) and in the extra test (AUC: 0.726, 95% CI: 0.556–0.865) (Fig. 3A). We performed a multivariate logistic analysis of NAC response in the training cohort to generate a nomogram to predict the results of pCR according to RCB index after NAC. Among the nine mutant genes and three clinical factors, MSH2, FOXP1 and luminal A were the three most important factors (Fig. 3B). Given the role of MP scoring, which has universal application in the clinic, we also tested the applicability of the model to MP scores and found that our model also predicted pCR and non-pCR well in MP score classification (Fig. 3C), and the nomogram showed that MSH2 still had the greatest importance (Fig. 3D).

Given that patients who achieved a pCR had better OS than patients who did not, we tested the predictive ability of the model on the prognosis. In total, 23 patients were lost to follow-up in the 192 enrolled patients from Shandong Cancer Hospital. The median follow-up time after surgery was 898 days (97 to 1765 days), and 28 of the 169 patients (17.6%) progressed. The results showed that the model also has a good predictive effect on the prognosis of NAC patients, and the predictive effect of the long-term prognosis was better than the short-term predictive effect (AUC at 1 year = 0.749 vs. AUC at 3 years = 0.830) (Fig. 3E). According to the median risk score, patients were divided into high-risk groups and low-risk groups. The DFS of the low-risk group was significantly higher than that of the high-risk group (*P* < 0.0001) (Fig. 3F).

### The role of ctDNA used to predict NAC response in dynamic monitoring

**Fig. 4:**
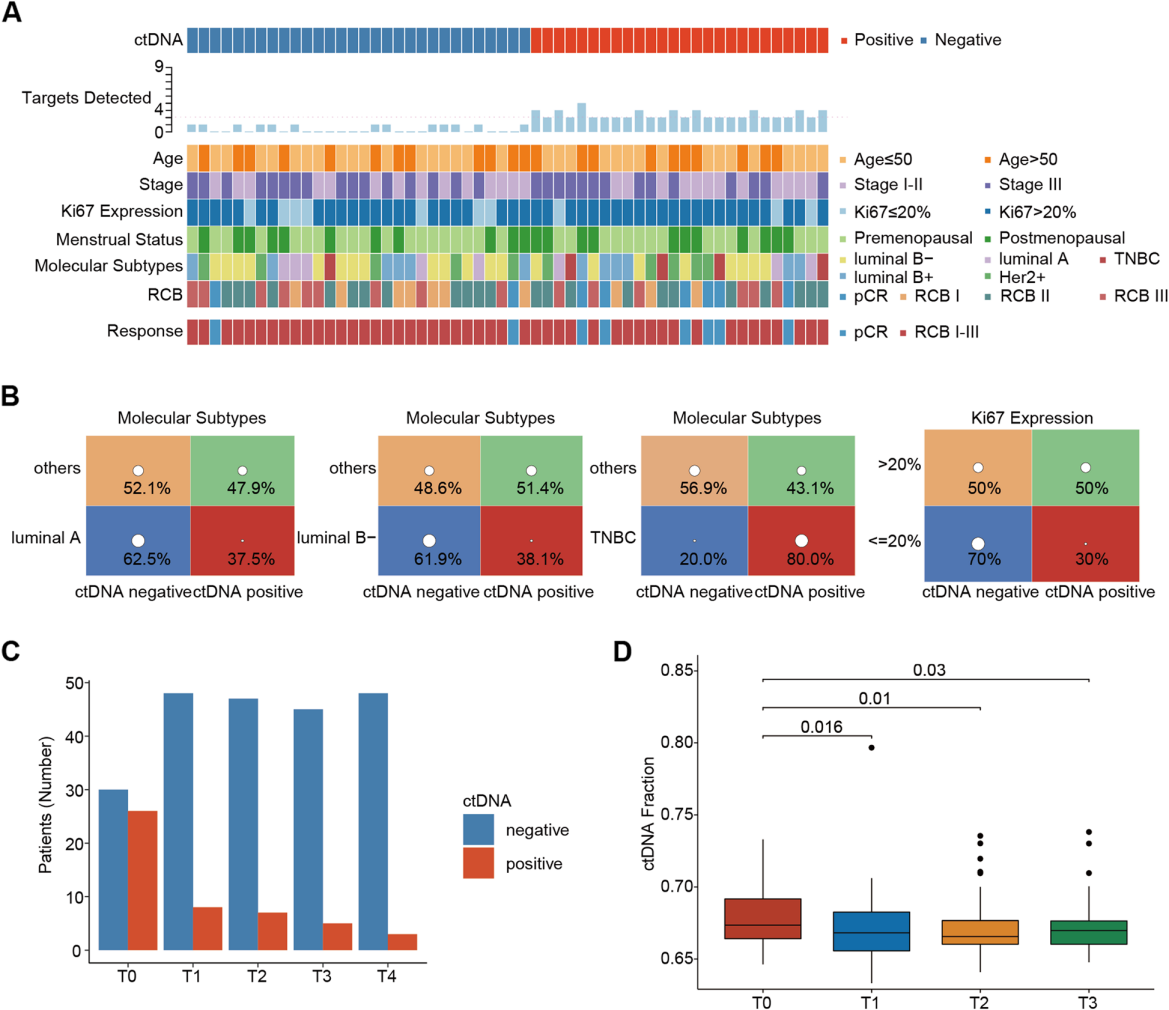
Mutation landscape of ctDNA. **A** Overview of ctDNA status, clinical characters, and response at baseline (T_0_). **B** Proportion of ctDNA-positive and ctDNA-negative patients at baseline (T_0_) according to clinical characteristics. **C** Proportion of ctDNA-positive and ctDNA-negative patients at different time points. **D** Comparing the difference of ctDNA fraction at different time points. *P* values were calculated using one-way analysis of variance

A personalized panel consisting of nine somatic mutation genes was selected to detect and analyze ctDNA from the tumor model factors for predicting NAC response. Fifty-six patients among all the 246 patients underwent ctDNA testing. two hundred sixteen blood samples were collected dynamically over the course of NAC (plasma samples were collected from 56 patients of T_0_ and T_1_, 54 patients of T_2_, and 50 patients of T_3_). A sample with at least two detectable somatic variations was considered positive for ctDNA (Additional file 1: Table S7). Before treatment (T_0_), 46% of patients were ctDNA positive (Fig. 4A). Patients with TNBC had a higher expression of positive ctDNA (80%) compared with other subtypes while luminal A and luminal B patients mainly had negative ctDNA (Fig. 4B). In addition, patients with low Ki67 status expressed negative ctDNA (70%) (Fig. 4B).

The ctDNA positivity rate decreased with the passage of time during NAC. In the entire population, ctDNA positivity gradually declined during NAC, from 46% before treatment (T_0_) to 14% before the 2^nd^ NAC cycle (T_1_), and it was 13% during intermediate evaluation (T_2_), and after NAC (T_3_), it dropped to 10% (Fig. 4C). Similarly, the ctDNA fraction also decreased with the passage of NAC time (Fig. 4D).

### The clearance dynamics of ctDNA reflected the NAC response

**Fig. 5:**
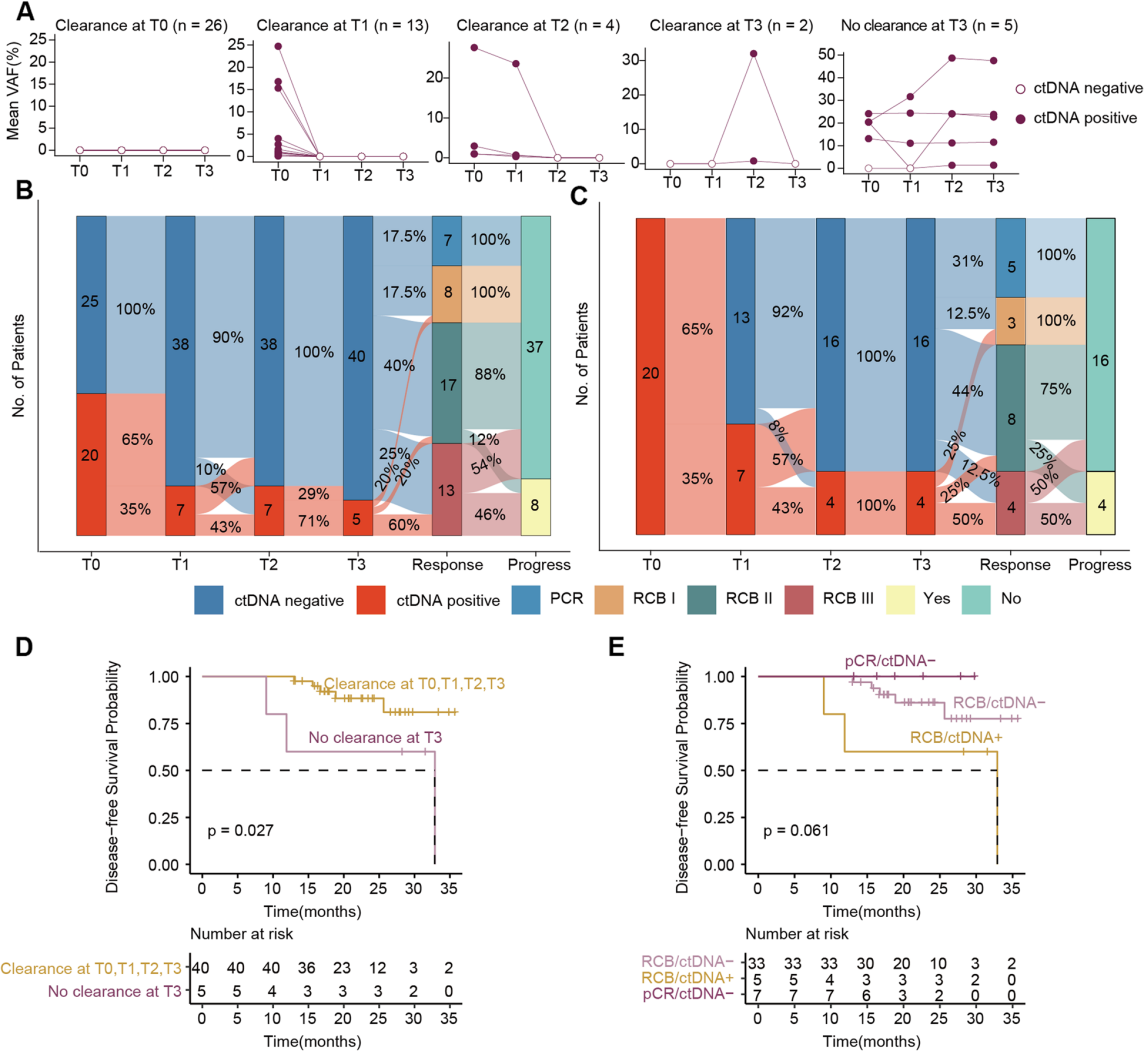
The association between the dynamic changes of ctDNA and DFS or response in the course of NAC. **A** Patients with complete ctDNA data for four time points (*n* = 50) were grouped according to the different patterns of ctDNA clearance or non-clearance. **B** Sankey plot showing the dynamic changes of patients with complete ctDNA data and DFS data (*n* = 45). **C** Sankey plot showing ctDNA dynamics in ctDNA-positive patients at T_0_. **D** DFS in ctDNA-cleared patients and non-cleared patients during NAC. **E** Kaplan–Meier analysis of DFS stratified based on ctDNA status after NAC (T_3_) and response to treatment, RCB means no-pCR

To investigate whether the dynamic change of ctDNA could related to the NAC response, we constructed five patterns based on the clearance dynamics of ctDNA expression in 50 patients who had complete data at all four time points, with cleared at all time points (T_0_, *n* = 26, 52%), cleared at T_1_ (*n* = 13, 26%), cleared at T_2_ (*n* = 4, 8%), cleared at T_3_ (*n* = 2, 4%), and patients who remained ctDNA positive after NAC (T_3_) (*n* = 5, 10%) (Fig. 5A). We identified 45 patients who had both survival data and ctDNA status at all four time points. The rate for positive detection of ctDNA decreased during the NAC, and the positive rate dropped from 44.4% (T_0_, 20/45) to 11.1% (T_3_, 5/45) (Fig. 5B). All the patients with pCR had undetectable ctDNA at T_2_ and T_3_ with no disease progression (Additional file 2: Fig. S2A, Fig. 5B). In contrast, patients with ctDNA-positive at T_2_ and T_3_ not achieved pCR (Fig. 5B). In addition, the patients with disease progression were mainly RCB-III (75%, 6/8) and RCB-II (25%, 2/8) (Fig. 5B).

To detect ctDNA dynamically for patients whose ctDNA is detected before the NAC (T_0_), we performed monitoring of the ctDNA expression at T_1_, T_2_, and T_3_. The positive rate of ctDNA gradually decreased as the NAC treatment with 35% (7/20) at T_1_ and 20% at T_2_ and T_3_ (4/20) (Fig. 5C). Among patients who did not clear ctDNA at T_1_, as many as 85.8% had residual disease at the time of surgery (6/7 non-pCR), while 69% of patients who cleared ctDNA at T_1_ had residual disease (9/13 non-pCR). At T_2_ and T_3_, in patients who did not have clear ctDNA, 100% had residual disease during surgery (4/4 non-pCR), while in patients who cleared ctDNA, 69% (11/16 non-pCR) had residual disease. The positive predictive value of ctDNA increased with treatment time (Additional file 2: Fig. S2B).

### Dynamic changes in ctDNA are significantly related to metastasis and recurrence

To assess whether ctDNA status was related to metastasis and recurrence, we analyzed the association with ctDNA dynamic pattern and DFS. Patients who did not clear ctDNA at T_3_ (*n* = 5) had a significantly higher risk of metastasis and recurrence than patients who cleared ctDNA at T_0_, T_1_, T_2_, and T_3_ (Fig. 5D, HR 4.61; 95% CI, 1.05–20.19, *P* = 0.027). Compared with patients who were ctDNA negative at T_0_ (*n* = 22), patients who were ctDNA negative at T_1_, T_2_, or T_3_ (*n* = 18) had a similar risk of metastasis and recurrence (Additional file 2: Fig. S3). Patients who had cleared ctDNA at T_1_, T_2_, T_3_ had longer DFS than patients who had not cleared ctDNA at T_3_ (Additional file 2: Fig. S3).

The clearance of ctDNA after NAC (T_3_) is related to the improvement of the survival rate. After NAC, patients were stratified according to pCR and ctDNA status (*n* = 45). Seven patients with pCR (100%) (all ctDNA negative) showed good DFS. Among patients who did not achieve pCR (*n* = 38), ctDNA positivity (*n* = 5) was related to worse DFS. The probability of recurrence differed between patients who failed to achieve pCR, being greater in RCB/ctDNA + groups compared with the RCB/ctDNA- group (Fig. 5E, HR 3.92; 95% CI, 0.9–17.02, *P* = 0.061).

### The chemotherapy prediction model integrating ctDNA status before NAC has a better prediction effect

**Fig. 6:**
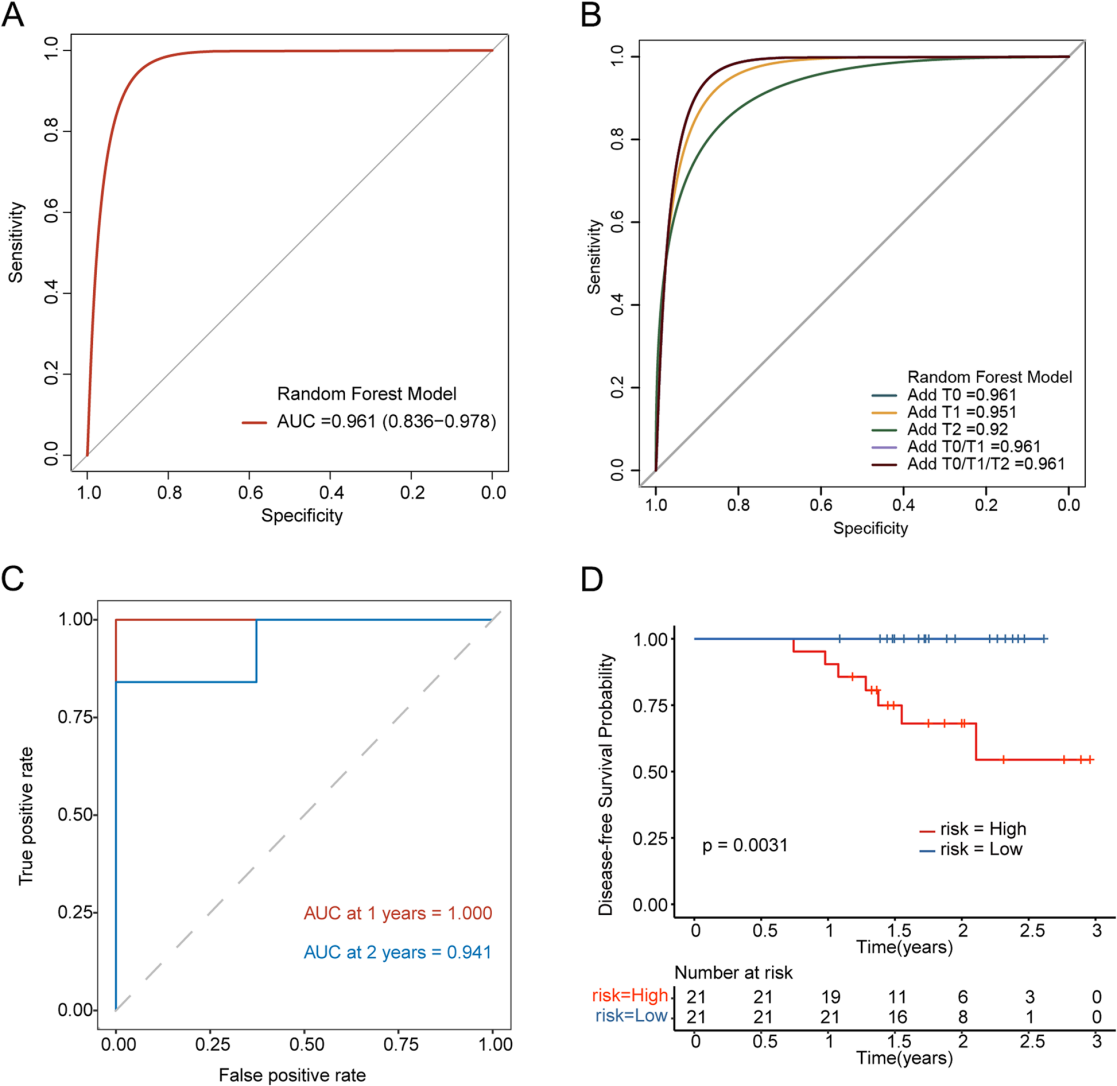
The prediction effect of pCR and the prognosis by a combination of the prediction model and ctDNA monitoring. **A** The pCR prediction determined by the chemotherapy predictive model constructed by combining the information from the established tumor prediction model (including DNA mutations and clinical factors), along with the information from ctDNA status. **B** Different chemotherapy predictive models are established using random forest based on the expression status of ctDNA at different time points of T_0_, T_1_, and T_2_. **C** The predictive effect for the chemotherapy predictive model on the prognosis of NAC patients. **D** Kaplan–Meier curves for patients in high- and low-risk group

To further improve the accuracy in predicting NAC response to further predict the pCR status of breast cancer patients after NAC, we calculated the probability of pCR and non-pCR of the sample through the established tumor prediction model, and then we combined it with the negative and positive status of ctDNA at different time points and used random forest to construct a chemotherapy model. Firstly, the status of ctDNA before NAC combined with the tumor prediction model has a better prediction effect of pCR with the AUC of 0.961 (Fig. 6A). We constructed a chemotherapy prediction model by combining tumor prediction models with ctDNA status at different time points (T_0_, T_1_, T_2_, T_0_ and T_1_, T_0_ and T_1_ and T_2_) in order to assess the impact of dynamic changes in ctDNA on prediction. It was found that the prediction model was compatible with ctDNA, and combined with ctDNA status at different time points had similar results for AUC (Fig. 6B, T_0_ = 0.961, T_1_ = 0.951, T_2_ = 0.92, T_0_/T_1_ = 0.961, T_0_/T_1_/T_2_ = 0.961). To clarify the impact of the chemotherapy prediction model on patients' prognosis, we analyzed the predictive effect of the DFS. It showed a better predictive effect (AUC at 1 year = 1.000, AUC at 2 years = 0.941) on DFS (Fig. 6C). The patients were divided into high-risk groups and low-risk groups according to the median risk score. The DFS of the low-risk group was significantly higher than that of the high-risk group (*P* = 0.0031) (Fig. 6D).

## Discussion

**Fig. 7:**
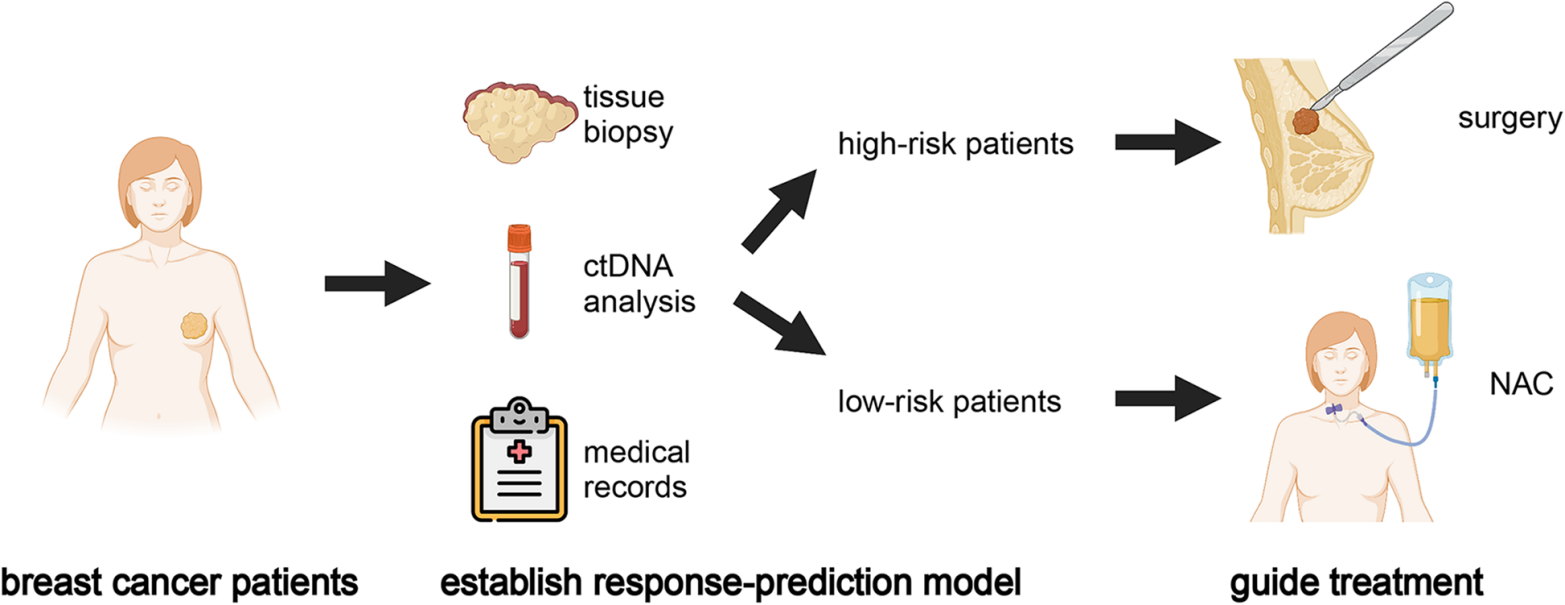
The pattern diagram guiding the clinical application of the therapeutic efficacy prediction model

In this study, we developed 2 prediction model for predicting the sensitivity of NAC. First, we constructed a tumor prediction model for predicting pCR based on the DNA mutations of tumor tissue and clinical information. Then, we analyzed the relation between the ctDNA in dynamic monitoring and the NAC response. Finally, we constructed the chemotherapy prediction model integrating ctDNA status before NAC and tumor prediction model that composed of 9-gene mutant in tumor, the clinical factors, and the ctDNA status. The chemotherapy prediction model is a good predictor for the efficacy in NAC to guide therapy, but also predicts the prognosis in DFS (Fig. 7).

The MP scoring and RCB index system are commonly recommended for pathological assessments after NAC for breast cancer. Compared with MP scoring, the RCB index system, first described in 2007, provides a comprehensive assessment of tumors for evaluating axillary lymph nodes and cell density in primary tumors [18]. RCB index system has become widely accepted to replace MP scoring to evaluate tumor regression due to the role in predicting long-term survival after NAC in breast cancer [26, 27]. Consequently, we used the RCB index to assess the chemotherapeutic response in the prediction models. In addition, our model based on the RCB index system also remained feasible with good sensitivity and specificity for the MP scoring system in the training set, validation set, and external validation set.

A mounting number of studies have suggested that patients with pCR after NAC have a better prognosis compared to those with residual disease [28–30]. The NSABP B-18 and NSABP B-27 trials reported that pCR was associated with improved prognosis after NAC in breast cancer [31, 32], which was also confirmed in subsequent cohort studies and meta-analyses [28, 33, 34]. Despite patients who obtain pCR after NAC having a better prognosis, the current pCR rate of patients receiving NAC is less than 50% [28, 35]. Our study is in line with these data, reporting a pCR rate of 25.1% (61/243) according to the RCB index system and 33.3% (81/243) by the MP scores in all 243 patients, suggesting that the majority of patients (non-pCR), there might not be benefit in survival from the routine use of NAC and experience unnecessary exposure to chemotherapy and delayed surgery treatment. Therefore, sensitive and specific markers are needed to distinguish between pCR and non-pCR patients after NAC.

We established a tumor prediction model by combining clinical factors (including molecular subtype and Ki67 status) and gene mutation information (MSH2, NOTCH4, PIK3CA, SETBP1, TP53, EGFR, FOXP1, IL7R, NFKB1), which had good sensitivity and specificity for predicting pCR in the training set, validation set, and external validation set. In our study, we observed that MSH2 and FOXP1 gene mutations and the luminal A subtype ranked in the top three in the importance of model predictors, consistent with previous studies. The MSH2 gene is part of the DNA mismatch repair system (MMR), which binds to DNA mismatches to initiate DNA repair. Previous studies have reported the importance of MSH2 in the role of resistance to chemotherapeutic drugs and causing progression to advanced stages in patients with NAC [36, 37]. FOXP1 is a member of the FOX transcription factor family which is associated with the development and prognosis in tumors. In a cross-sectional study of breast cancer, an analysis of stage I to III breast cancer patients who received NAC from 2018 to 2019 found that, in response to treatment, there was a significant association between complete response and FOXP1 (*p* = 0.01) [38]. The insensitivity of the luminal A subtype of breast cancer to NAC has been reported. In the I-SPY 1 trial, it was found that the most insensitive subtype to adjuvant chemotherapy or NAC was luminal A, with a pCR rate of only 9% [39]. Our results provided more evidence for the predictive value of incorporating gene mutant signatures into clinical risk stratification.

Additionally, considering the high concordance rate of somatic mutations between ctDNA and tumor DNA, we analyzed ctDNA using a unique personalized panel from the constructed model [40]. Our study tracks up to 9 patient-specific somatic variants at the same time for offering a performance in the heterogeneity of a patient’s tumor as previously reported [41]. Nevertheless, several limitations were associated with this method. For example, newly emergent somatic variants which presented during tumor evolution in response to NAC treatment were usually not detectable.

Our data found that a significant reduction in pre-operative ctDNA level during the NAC could predict the pCR, suggesting that dynamic ctDNA monitoring may be helpful in tailoring the treatment regimen of NAC. Among all patients with ctDNA positive in T_0_, the positive rate of ctDNA gradually decreased during the NAC treatment with 35% at T_1_ and 20% at T_2_ and T_3_. The patients with pCR after NAC were all patients who tested negative for ctDNA at T_2_ and T_3_. At T_2_ and T_3_, no ctDNA-positive patient achieved pCR and the main grade was RCB-III. These results suggest that the ctDNA status of patients who experienced at least one chemotherapy cycle may predict the response of NAC. Furthermore, the ability to predict the response of NAC became more accurate with increasing numbers of cycles of chemotherapy, and the time points of T_2_ (during intermediate evaluation) and T_3_ (after the end of NAC but before surgery) may be taken to obtain blood samples for ctDNA analysis.

Interestingly, we demonstrated the prognostic value of ctDNA status, therefore, might probably act as a promising predictor of metastasis and recurrence in breast cancer patients with NAC. The patients with ctDNA-positive at T_3_ had significantly worse DFS compared with the patients with ctDNA-negative. We observed similar results after stratifying according to pCR and ctDNA status after NAC. The patients who achieved pCR with ctDNA negative had the best outcomes, and patients who failed to achieve pCR with ctDNA positive had the worst outcomes. In patients who did not achieve pCR, ctDNA negative was significantly associated with better DFS than ctDNA positive. Our results are consistent with recent studies [41]. The presence of ctDNA reflects the presence of metastatic tumor burden, and the presence of elevated ctDNA levels predicts disease progression [42, 43].

Considering the importance of ctDNA to predict patient response to NAC [44], ctDNA status was added as a categorical factor to refine the tumor predictive model [45]. We constructed the chemotherapy predictive model using the ctDNA status before NAC combined with the tumor predictive model. This new chemotherapy predictive model effectively reflected the sensitivity of NAC and predicted the patient’s prognosis for distinguish high- and low-risk patients. It is vital to predict and judge the response to the NAC for subsequent treatment strategies. Research has demonstrated that the high residual cancer burden after NAC in breast cancer signifies a poor prognosis. Additionally, these non-responders can benefit from additional adjuvant chemotherapy. As shown in the recently published CREATE-X trial, capecitabine was used for 6 months in TNBC patients who did not obtain pCR, leading to an improved overall survival rate and DFS [46, 47]. Our results may have important implications for predicting the response of NAC and rational treatment guidance.

In this study, there are some limitations. Firstly, the tumor model constructed by tumor DNA mutant and clinical information has been internally validated and externally validated. However, the chemotherapy model constructed by ctDNA status has not been further validated due to the limited number of patients. Only 56 had the ctDNA analysis and there was no external independent verification. Second, the follow-up period was limited to 2–3 years, and a long-term follow-up is needed to confirm our results. Therefore, the results will be further verified by a prospective cohort with a larger sample size and longer follow-up time.

## Conclusions

The focus of this study was to construct a prediction model in the neoadjuvant setting. Multiscale approaches that integrate clinical and genomic DNA mutations of tumors were used to construct the predictive model to predict response to NAC and prognosis. In addition, given the prognostic value of ctDNA in breast cancer patients, we included pretreatment ctDNA levels in the predictive model. The model integrating ctDNA may have a more reliable predictive efficacy, but a larger cohort of patients is needed to validate our findings.

### Supplementary Information

**Additional file 1: sTable 1.** The pCR rate in different molecular subtype. **stable 2.** Patient Clinical Characteristics (Train and Test Datasets). **stable 3.** Patient Clinical Characteristics (Extra Test Datasets). **stable 4.** Patient SNV Characteristics (Train and Test Datasets). **stable 5.** Patient CNV Characteristics (Train and Test Datasets). **stable 6.** significant SNV and CNV. **stable 7.** Patient ctDNA Characteristics.

**Additional file 2: sFig. 1.** Comparing the differences in the performance of predicting pCR with different clinical factors, SNV and CNV characters. **sFig. 2.** (A) Sankey plot showing the differences in patients ctDNA cleared at different time points (T_1_, T_2_, T_3_). (B) Sankey plot showing the differences in patients with positive ctDNA at different time points (T_1_, T_2_, T_3_). **sFig. 3.** Kaplan–Meier analysis of DFS stratified based on ctDNA status during NAC. **sFig 4.** Overall algorithm flowchart for the predictive model construction.

## Data Availability

The raw data of the datasets generated during the current study could be achieved from the public accession GSA-Human database (HRA004909), and the processed data are available from the corresponding author on reasonable request.

## Abbreviations

ACC: Accuracy
AJCC: American Joint Committee on Cancer
CNV: Copy number variation
ctDNA: Circulating tumor DNA
DFS: Disease-free survival
FFPE: Formalin fixation and paraffin embedding
MP: Miller-Payne
MRI: Magnetic resonance imaging
NAC: Neoadjuvant chemotherapy
pCR: Pathological complete response
ROC: Receiver operating characteristic
SNV: Single nucleotide variants
TMB: Tumor mutation burden
TNBC: Triple-negative breast cancer
VAF: Variant allele frequency
